# Supplementary material for: Identification of photoperiod-induced specific miRNAs in the adrenal glands of Sunite sheep (Ovis aries)
Source: Front Vet Sci. 2022 Jul 22;9:888207. doi: 10.3389/fvets.2022.888207 (PMC9354845; doi:10.3389/fvets.2022.888207)
Supplement: Supplementary file 1 [file Data_Sheet_1.ZIP › Supplementary file/miRDeep2 file.zip!/miRDeep2 file/Novel_Structure/SPLP42A3/Novel_4.pdf]

Provisional ID	:	NC_019476.2_30669
Score total	:	0.1
Score for star read(s)	:	-1.3
Score for read counts	:	0
Score for mfe	:	-0.1
Score for randfold	:	1.6
Score for cons. seed	:	
Total read count	:	105
Mature read count	:	105
Loop read count	:	0
Star read count	:	0

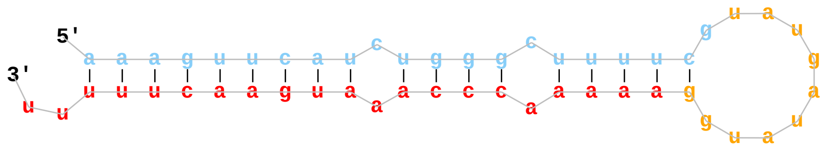

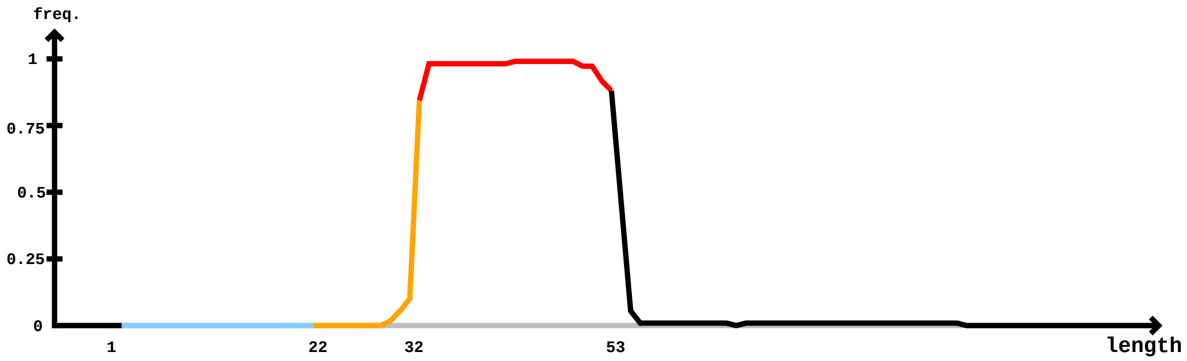

Star Mature

```
5'- accaaaaaguucaucugggcuuuucguaugauauggaaaaacccaaaugaacuuuuugaccaacccaauaccuauuuguaugcuucuccugcucaguugcucagugaugucug   -3'   exp
    ..((((((((((((.((((.(((((..........))))).)))).))))))))))))........((((......))))((.((....((......)).....)).))...      reads     mm     sample
    .................................uggaaaaacccGaaugaacu............................................................      2         1      seq
    ..................................ggaaaaacccaaaugaacuuu..........................................................      1         0      seq
    ..................................Ugaaaaacccaaaugaacuuu..........................................................      1         1      seq
    ..................................ggaaaaacccaaaugaacuuuu.........................................................      2         0      seq
    ...................................gaaaaacccGaaugaacuuu..........................................................      1         1      seq
    ...................................gaaaaacccaGaugaacuuu..........................................................      2         1      seq
    ...................................gaaaaacccGaaugaacuuuu.........................................................      1         1      seq
    ...................................gaaaaacccaGaugaacuuuuu........................................................      1         1      seq
    ....................................aaaaacccGaaugaacuuu..........................................................      1         1      seq
    ....................................aaaaacccaGaugaacuuuu.........................................................      1         1      seq
    ....................................Gaaaacccaaaugaacuuuuu........................................................      3         1      seq
    ....................................aaaaacccaGaugaacuuuuu........................................................      27        1      seq
    ....................................aaaaacccaaaugaacuuuuuA.......................................................      2         1      seq
    ....................................Caaaacccaaaugaacuuuuug.......................................................      3         1      seq
    ....................................aaaaacccGaaugaacuuuuug.......................................................      2         1      seq
    ....................................Gaaaacccaaaugaacuuuuug.......................................................      6         1      seq
    ....................................aaaaCcccaaaugaacuuuuug.......................................................      1         1      seq
    ....................................aaaaacUcaaaugaacuuuuug.......................................................      24        1      seq
    ....................................aaaaGcccaaaugaacuuuuug.......................................................      2         1      seq
    ....................................aaaaacccaaaugaacuuuuug.......................................................      8         0      seq
    ....................................aaaaacccaaaugaacuuuuuga......................................................      1         0      seq
    .....................................aaGacccaaaugaacuuuuu........................................................      1         1      seq
    .....................................aaaacUcaaaugaacuuuuug.......................................................      10        1      seq
    .....................................aaaacccaaaugaacuuuuuga......................................................      4         0      seq
    ..............................................augaacuuuuugaccaacccaaA............................................      1         1      seq
    ......................................................................ccuauuuguaugcuucuccugcu....................      1         0      seq
```
